# Ligature Mark: Crime or Suicide?

**DOI:** 10.3390/ijerph192114221

**Published:** 2022-10-31

**Authors:** Gelsomina Mansueto

**Affiliations:** 1 Department of Advanced Medical and Surgical Sciences, University of Campania “Luigi Vanvitelli”, 80138 Naples, Italy; gelsomina.mansueto@unicampania.it; mansueto.g@inwind.it; 2 Clinical Department of Laboratory Services and Public Health-Legal Medicine Unit, University of Campania “Luigi Vanvitelli”, Via Luciano Armanni 5, 80138 Naples, Italy; 3 Pathology Section, Federico II University, Via Sergio Pansini 5, 80131 Napoli, Italy

Identifying the morphological findings of vitality is crucial when determining if a lesion was inflicted in life or postmortem. It is difficult to place all vitality criteria in a broad context when doubt arises between a homicidal death and a suicidal death. The differential diagnosis between total hanging and partial hanging, strangulation and throttling represents a flotant and obscure limbo, especially in unusual finds. The forensic pathologist is not always certain, but is increasingly hindered in their diagnosis by the need for on-the-spot investigation and the first external cadaver examination. Often, in fact, the first erroneous reconstruction of the facts is misled by unusual situations, and a non-coincident pathological diagnosis is not considered. Therefore, collaboration between different specialists remains delicate and necessary.

Currently, as evidenced by the literature that we reviewed in a previous paper [1], researchers have not discovered many specific molecular and immunohistochemical markers or valid uses for them in the easier diagnosis of viability in ligature marks [1]. The only criteria that remain firmly established are represented by microscopic findings that must be framed in a wider context through the expert eyes of a pathologist. My opinion is that only in this way can we provide useful tips to those who come across particular cases on a daily basis that require experience and support for legal purposes.

Before even relying on hypothetical immunohistochemical or molecular markers, it is necessary to carry out careful external examination of the corpse, evaluating multiple differential diagnoses, even when the inspection is completely directed towards suicide or homicide. In surgical pathology, the same attention is necessary for macroscopic examination of all the organs and all the anatomical structures, supported by the traceability of information that is useful for subsequent correct sampling; this should guarantee correct final preparation of slides that are useful for microscopic observation, for interpretation of the data and for the final diagnosis. A good description of a skin sulcus associated with a ligature, for example, is rarely read during the work routine, and just as seldom do we read a good histological report. Microscopic data can sometimes have different meanings if interspersed among people with different constitutional types and in different contexts that are affected by multiple variables. It should be emphasized that in pathological anatomy, and even more so in forensic pathology, although the histological criteria are diagnostic, they must be framed in a general systemic complex considering many variables such as the hanging furrow, the possibility of different constitutional types, unusual means of harm, etc.

Therefore, the correct localization of the sulcus specimen, its orientation on the corpse and all the structural characteristics assume considerable importance. Understanding how the serial histological sampling was performed is useful in determining the exact topography by which the lesion acted, especially when it is not typical and when it could simulate an act other than hanging. Without any doubt, the formulation of a good histological report is fundamental in a forensic investigation and cannot be separated from careful macroscopic examination.

Macroscopy and microscopy, supported by information, together produce a conclusive diagnosis. For skin samples, the relief of the three major diameters (longitudinal, transverse and deep) and the description of the surface of the cutaneous and subcutaneous portion and of everything that differs from normal skin can take on a different meaning; for example, on the basis of constitutional type, everything should be carefully reported, especially when in specific cases, such as hanging, few signs of vitality are observed. To be more exhaustive, if the skin or subcutaneous striated muscle tissue is poorly represented for the typical anatomical site, it is obvious that an erythrocyte extravasation will affect minor, partial and different planes and may not even be present. Furthermore, for example, the anterior region of the neck—the site of organs such as the thyroid and larynx—has less-represented striated muscle tissue than the anterolateral ones; in the latter, the muscular component is thicker, with an oblique course of the fibers, and there are vascular structures of greater caliber belonging to the vascular-nerve bundle. For such variability in the quantitative and structural representation of skin and soft subcutaneous tissues, the identification, localization and estimation of histological vitality findings certainly cannot be superimposed in all cases. Furthermore, it is necessary to consider the variability of the pressures exerted by the means of injury.

Each diagnosis should be interleaved in a broader context. Everything is very important and unrepeatable, since the sample, after processing, no longer exists in its entirety, which could compromise a good microscopic and conclusive diagnosis.

Generally, in routine work activities, everything is reduced in a minimalist way to the so-called “skin sulcus”. We lose sight of the fact that pathological diagnosis made of macroscopic and microscopic observation together, on the basis of consolidated objective morphological criteria, requires knowledge and interpretation of the data, unlike some laboratory tests such as the evaluation of cholesterolemia, which is now carried out using automated devices.

A single datum cannot provide a diagnosis. In the context of death by violent asphyxiation, it is necessary to highlight the possible differential diagnoses, especially between homicidal or suicidal hanging. It is necessary to consider the vitality findings of the sulcus, intercalating them at the sampling site and considering the presence of a greater or lesser amount of fat, muscles and more. Furthermore, it is also necessary to consider the morphological aspects of other nearby and/or distant organs, structures and tissues (e.g., the thyroid, larynx, lungs, etc.) [2,3,4].

It is difficult to frame situations such as those in which the corpse is found in a particular position touching the ground, that is, in partial or incomplete suspension. In this case, the challenge centers on the question of whether hanging could represent an act of suicide or not and arises from the fact that the body is not completely suspended [4]. In support of this, many studies have shown, on a large series of hangings that: (i) In partial self-suspensions, the appearance of the ligature mark can be atypical; there may not be a complete ligature mark around the neck or an upward slope, or there may not be a ligature mark at all; (ii) The pressure required to occlude the jugular veins or carotid arteries is less than the weight of an average adult head and can be reached during total or partial suspension; (iii) Petechiae and bruising can be present in over 50% of victims of suicidal and incomplete hanging (they appear when venous pressure above the level of the ligature increases, causing vessels to rupture and blood to leak into surrounding tissues; this occurs commonly in the head and neck area and can affect both the skin and the mucous membranes); (iv) If there is incomplete occlusion of the vessels of the neck or trachea, as is possible in partial suspension, the victim can wrestle and may scratch or claw at the ligature, hitting nearby objects, or experience seizure-like activities; (v) Pulmonary findings are important for differential purposes. Emphysema, a sign of mechanical asphyxia, is considered a sign of vitality in incomplete self-suspension, with an evaluation of lung extension almost equal to that of drowning [4,5,6]. A good histological examination of all organs is required to confirm or refute the first diagnostic hypothesis carried out during the inspection and autopsy by the coroner.

The overall anatomopathological evaluation of the vitality of a sulcus therefore constitutes a complex and demanding diagnostic act; it must be supported by many data, and also verified in a range of possible differential diagnoses, in cases where the question is divided between homicide and suicide. In particular and unusual cases, such as in partial self-hangings/strangulations, the histological criteria are more often discontinuously present due to the different intensities of the forces exerted by the means of injury at different points on the skin; they sometimes presuppose hesitation or the uncontrolled movement of the victim. In these cases, the signs of vitality are not quantitatively superimposable, but rather, are lower than those observed in complete and typical hangings, and in strangulation. In the latter cases, the force of the injury is exerted in a rapid and violent way with a more regular and limited distribution of pressure, which is often responsible for bone fractures or the complete rupture of neck vessels [4]. It should also be noted that some experts of forensic pathology report that: in cases of hanging, the external pressure is disproportionate from case to case, and can act on the neck at different levels and in different areas in an unequal way [7]; the intensity and distribution of the constricting forces, together with the completeness of the body’s suspension, can determine a wide range of possible histological findings [7,8]; the type of loop, the elasticity and width of the ligature and the type of knot can influence the extent and frequency of neck injuries [9,10,11,12]; stiff and narrow ligatures produce deeper and sharply demarcated marks and logically produce a more localized force effect against neck structures, while wide, looser ligatures tend to create less noticeable ligature marks and, due to their distribution being more of a planar than a pressure load, less severe injuries [13]. The literature also reports a statistically more significant association between the onset of bleeding and the completeness of body suspension, as well as a significant association between the onset of bleeding and the location of the ligature knot on the neck, while it does not demonstrate a significant association between hemorrhages in the muscles (sternocleidomastoid) and various parameters, including body weight; therefore the hemorrhage remains a non-contributory value in quantitative terms [14].

On the basis of this brief scientific review, it is easy to understand how each element of an evaluation, inserted in the right context, is useful for the diagnosis of vitality; the criteria for this still remain erythrocytic extravasation, epidermal excoriation, fragmentation of the connective tissue and folding of the epithelium towards the edges of the sulcus [15], and not semi-quantitative or percentage evaluation of the number of red blood cells. In fact, to date, no work has set a percentage or semi-quantitative numerical value (e.g., low, moderate or severe) above which the sulcus must be considered vital, just as no work has highlighted an immunohistochemical or biological marker which can be considered the panacea.

Retrospective studies have reported many strange and unimaginable means of injury, as well as positions in which corpses were found. Tulapunt et al., reported that out of 245 cases of hanging, 21% were found in a sitting position, 4% on their knees and 1.6% lying down. They are small percentages, but they exist, and are therefore indicative and useful for an approach to differential diagnoses. The same authors report the importance of anatomopathological morphological aspects of different districts and organs, such as pulmonary edema, the presence of facial bruises, peripheral congestion, etc., as important elements that favor a diagnosis of partial rather than total hanging [5].

The evaluation of suicidal suspension is complex; it requires the integration of different data and a good autopsy with the study of different organs, especially when elements such as microfractures are not macroscopically detectable or when erythrocyte extravasations that are not macroscopically evident involve organs such as the thyroid and the larynx.

It is now clear that in mechanical asphyxia, the diagnostic morphological aspects can be found in the mucous membranes, as authoritative authors describe; in the lung, which certainly represents an estimate of possible agony; and in many other organs. In fact, it has been observed that emphysema is a sign of vitality in incomplete suicidal hanging with an evaluation of lung extension almost equal to that of drowning [6], while inflammation with activated macrophages is typical of the hypoxia damage that occurs in prolonged asphyxia, and therefore in agony (at least 25 min) [16,17].

It should be emphasized that a differential diagnosis for the purpose of justice cannot be based solely and exclusively on the ligature mark; moreover, such a mark could be absent in incomplete suicidal hangings. It should equally be emphasized that during an inspection, only one hypothesis must be posed which can subsequently be validated, completed or modified following the outcome of the histological examination.
